# Microbiome in *Cladonia squamosa* Is Vertically Stratified According to Microclimatic Conditions

**DOI:** 10.3389/fmicb.2020.00268

**Published:** 2020-02-25

**Authors:** Hyun-Ju Noh, Yung Mi Lee, Chae Haeng Park, Hong Kum Lee, Jang-Cheon Cho, Soon Gyu Hong

**Affiliations:** ^1^Division of Polar Life Sciences, Korea Polar Research Institute, Incheon, South Korea; ^2^Department of Biological Sciences, Inha University, Incheon, South Korea

**Keywords:** Antarctica, lichen, bacteria, fungi, microalgae, microbiome

## Abstract

Lichens are miniature ecosystems that contain fungi, microalgae, and bacteria. It is generally accepted that symbiosis between mycobiont and photobiont and microbial contribution to the ecosystem support the wide distribution of lichens in terrestrial ecosystems, including polar areas. The composition of symbiotic components can be affected by subtle microenvironmental differences within a thallus, as well as large-scale climate differences. In this study, we investigated fine-scale profiles of algal, fungal, and bacterial compositions through horizontal and vertical positions of the Antarctic lichen *Cladonia squamosa* colonies by next-generation sequencing of the nuclear large subunit rRNA gene (nucLSU) of eukaryotes and the 16S rRNA gene of bacteria. Apical parts of thalli were exposed to strong light, low moisture, and high variability of temperature compared with basal parts. Microbial diversity increased from apical parts to basal parts of thalli. *Asterochloris erici* was the major photobiont in apical positions of thalli, but other microalgal operational taxonomic units (OTUs) of Trebouxiophyceae and Ulvophyceae were major microalgal components in basal positions. Photochemical responses of algal components from apical and basal parts of thalli were quite different under variable temperature and humidity conditions. Several fungal OTUs that belonged to Arthoniomycetes and Lecanoromycetes, and diverse bacterial OTUs that belonged to *Alphaproteobacteria*, *Acidobacteria*_Gp1, and candidate division WPS-2 showed a clear distribution pattern according to their vertical positions within thalli. The overall lichen microbiome was significantly differentiated by the vertical position within a thallus. These results imply that different microclimate are formed at different lichen thallus parts, which can affect microbial compositions and physiological responses according to positions within the thalli.

## Introduction

Lichens are symbiotic organisms that are majorly composed of lichenized fungi (mycobiont) and green algae and/or cyanobacteria (photobiont) (De Bary, 1879; Nash, 2008). It has long been thought that a single photobiont forms a symbiotic relationship with one mycobiont, but different algal species have been detected in a single lichen thallus in diverse lichen species in recent studies (Guzow-Krzeminska, 2006; Ohmura et al., 2006; Piercey-Normore, 2006; Casano et al., 2011; Park et al., 2015). Two *Trebouxia* species were found in *Ramalina farinacea*, and they responded differently to temperature and irradiance (Casano et al., 2011). Moreover, differentiated *Trebouxia* and *Asterochloris* compositions along the laciniae of *R. farinacea* suggested that localization of photobionts can be affected by different microenvironmental conditions (Moya et al., 2017). Detection of different algal species in a single lichen thallus and varying responses to different microenvironments have led to the hypothesis that switch of algal species or the presence of different algal species can play roles in adaptation of lichens to changing environments (Blaha et al., 2006; Guzow-Krzeminska, 2006; Ohmura et al., 2006; Piercey-Normore, 2006; Casano et al., 2011; Park et al., 2015; Moya et al., 2017).

In addition to the mycobiont and photobiont, diverse microorganisms, such as lichen-associated fungi (LAF) and lichen-associated bacteria, have been revealed by culture-dependent and molecular approaches (Lawrey and Diederich, 2003; González et al., 2005; Liba et al., 2006; Cardinale et al., 2008; Grube et al., 2009; Bates et al., 2012; Hodkinson et al., 2012; Lee et al., 2014; Biosca et al., 2016; Park et al., 2016). With the dominance of Ascomycota and Basidiomycota in LAF and *Alphaproteobacteria* in bacterial communities across diverse lichen species, lichen-associated microbial compositions are known to be largely influenced by various environmental factors (Grube and Berg, 2009; Bates et al., 2011; Cardinale et al., 2012; Hodkinson et al., 2012; U’Ren et al., 2012; Beck et al., 2014; Park et al., 2015, 2016; Wang et al., 2016). For example, lichen-associated fungal communities varied according to the host species (Beck et al., 2014; Park et al., 2015; Fernández-Mendoza et al., 2017; Banchi et al., 2018), climate conditions (U’Ren et al., 2012), seasonal changes of environmental conditions (Beck et al., 2014), geographic location, and altitude (Wang et al., 2016). Bacterial compositions in lichens were influenced by host species (Bates et al., 2011), age and sun exposure (Cardinale et al., 2012), substrate type and growth form (Park et al., 2016), and photoautotrophic symbiont and geography (Hodkinson et al., 2012). These results imply that microbial compositions in lichen thalli are affected by surrounding biotic and abiotic factors.

Microbiomes of lichens are also known to be influenced by microenvironments in a single lichen thallus. Microenvironments, such as differences in direction and exposure to sunlight, were suggested to cause differentiation in fungal communities (Unterseher et al., 2007; Beck et al., 2014). In the case of foliose lichen *Xanthoparmelia*, the bacterial community was quite different depending on the horizontal position (Mushegian et al., 2011). The center of individual lichen, which is exposed to longer periods of sunlight than the edge and retains vertical reproductive structure named isidia that was deduced to provide bacteria with differential microenvironment in light and moisture availability, showed diverse and consistent bacterial diversity. Another research on *Cetraria islandica* and *Cladonia arbuscula* has shown that bacterial communities of young and old thalli were significantly different (Cardinale et al., 2012). Younger upper part was majorly composed of *Alphaproteobacteria* while older basal part contained increased abundance of *Actinobacteria*, *Gammaproteobacteria*, and *Betaproteobacteria*. It was also shown that sun exposure affected bacterial community in the same study. Although the importance of microenvironment as a determinant of lichen microbiome has been revealed, very little information on microbial communities according to horizontal and vertical positions within a lichen is available. The lichen microbiome differentiation according to variable microenvironments can give valuable insights into how lichens adapt even to subtle microenvironmental changes and how diverse components of lichen microbiome interact with each other.

Among diverse species of the genus *Cladonia*, which are widespread on King George Island, Antarctica (Stenroos, 1993; Osyczka and Olech, 2005; Kim et al., 2006; Lee et al., 2008), *Cladonia squamosa* (Scop.) Hoffm., which usually grows on mosses such as *Chorisodontium aciphyllum*, *Polytrichum strictum, Andrea gainii*, and *Sanionia uncinata*, forms colonies composed of slender vertical thalli (Øvstedal and Smith, 2001; Osyczka and Olech, 2005). The surface of an upright secondary thallus, called a podetium, is surrounded by squamules of grayish-green or brownish-green color (Stenroos et al., 2002; Osyczka and Olech, 2005). Apical parts of thalli are considered to be exposed to direct sun light and dry air, and they produce secondary metabolites like melanin to protect themselves from oxidative stress (Legaz et al., 1986; Gauslaa and Solhaug, 2001; Cocchietto et al., 2002). In contrast, basal parts of thalli are hidden from direct sunlight and can maintain relatively stable moisture owing to the water supply from the substrate, similar to other lichens living on mosses and liverworts (Maass and Yetman, 2002; Colesie et al., 2012; Cornejo and Scheidegger, 2016). Morphological characteristics of *C. squamosa* led us to consider *C. squamosa* as a good model organism to investigate the effects of microenvironments on lichen microbiome composition. In this context, we investigated the microalgal, fungal, and bacterial compositions along the thalli of *C. squamosa* and tried to identify relationships between microbiome composition and three-dimensional position within the lichen colony.

## Materials and Methods

### Monitoring of Environmental Conditions

Environmental conditions of sampling site were monitored using HOBO loggers and sensors (Onset, MA, United States). Temperature, relative humidity and photosynthetically active radiation (PAR) were measured at the height that are exposed to the atmosphere without snow cover (80 cm aboveground) and at nearby the lichen thallus (10 cm aboveground). Temperature and water content were measured at the substrate just below the lichen thallus (5 cm underground). Temperatures at heights of 80 and 10 cm aboveground and at a depth of 5 cm underground were monitored for 1 year from February 19, 2014 to February 28, 2015. Relative humidity at 80 and 10 cm aboveground and soil moisture at 5 cm underground were monitored from February 19 to March 21, 2014. PAR was monitored at 80 and 15 cm aboveground for 1 year from February 19, 2014 to January 20, 2015.

To measure light penetration through the *C. squamosa* colony, a Mini-PAM PAR sensor (Walz, Germany) was positioned at apical, middle, and basal parts of the lichen colony and illuminated with 162 μmol/m^2^/s of blue and red light (DYLED44C, Dyne Bio, Korea). Measurements were repeated five times at each position. Variability of the water content of the thallus according to water content in the substrate was measured under conditions of continuous water supply and air-drying of the lichen colony. Dried *C. squamosa* colony with substrate moss was placed on the paper towel and water content sensor was installed in the substrate moss. Water was supplied to the paper towel until the substrate was fully hydrated. After then it was air-dried for 28 h. Three lichen thalli were sampled from the colony for every 1 h during hydration and dehydration. Water content in the apical, middle, and basal parts of lichen thalli was measured by determining the difference between wet and dry weight of the thallus after drying at 60°C for 24 h. Average value of measurements for three thalli was presented.

### Sample Collections and Processing

**FIGURE 1:**
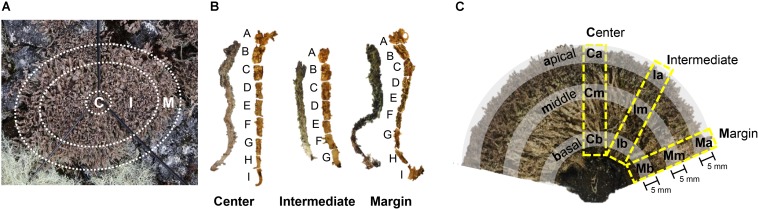
Sample preparation of *Cladonia squamosa* for microbiome analyses. **(A)** Top view of the lichen colony. Thalli were collected from central (C), intermediate (I), and marginal (M) positions. Central position was within 2 cm from the center of the colony and marginal position was within 2 cm from the edge of the colony. **(B)** Each thallus was sectioned by 3–5 mm interval for fine-scale microbiome analysis. **(C)** Side view of the sectioned lichen colony. Lichen thalli were collected from the Central, Intermediate, and Marginal positions in the colony and subsamples were prepared from the apical, middle, and basal parts of each thallus for statistical analysis.

Specimens of *C. squamosa* were collected from three colonies on King George Island, Antarctica (S 62°14′26″, W 58°44′32″), in January 2014. Colonies were divided into central, intermediate, and marginal positions (Figure 1A). The central position had a diameter of 2 cm and the marginal position was within 2 cm from the edge of the colony. The intermediate position was between the central and marginal positions. Thalli from each position in three colonies were fixed in 100% ethanol immediately after collection. Lichen samples were transported to the laboratory in Korea at −20°C. The thalli were dissected under a microscope to remove contaminants, such as liverwort, moss debris, and soil.

To investigate the microbiome composition along the thallus, each thallus from central, intermediate, and marginal positions of a colony was sectioned at 5 mm intervals to create seven or nine subsamples depending on the length of the thallus (Figure 1B). Each subsample was washed with 0.85% NaCl by vortexing for 5 min and removing supernatant after spin down. The washing procedures were repeated three times, as described by Lee et al. (2014).

To confirm findings from the finely sectioned thalli from one colony, microbiomes from three colonies of *C. squamosa* were analyzed. In this case, thalli were taken from the central, intermediate, and marginal positions of different colonies, and each thallus was sectioned into three parts, apical, middle, and basal parts, as illustrated in Figure 1C. Apical and basal parts were 5 mm long from the tip or the base of thallus, respectively. A middle part was taken from the thallus where the color changes from dark brown to light brown or light green. Each part was labeled according to the horizontal positions of thalli in the colony (**C**entral, **I**ntermediate, and **M**arginal) and vertical positions in each thallus (**a**pical, **m**iddle, and **b**asal) as illustrated in Figure 1C. Three thalli from one colony and two thalli each from two additional colonies were analyzed for each position. Three colonies were located within a radius of 1.5 m.

### DNA Extraction, PCR Amplification, and Sequencing

Samples were ground into fine powder using TissueLyser II (Qiagen, Germany) after freeze-drying and DNA was extracted using FastDNA spin kit for soil (MP Biomedicals, CA, United States) according to the manufacturer’s instruction. To investigate the diversity of eukaryotic microorganisms, the nuclear large subunit rRNA gene (nucLSU) was amplified with eukaryote universal primers, LSU26f and LSU657r, as described in a previous study (Park et al., 2015). The bacterial 16S rRNA gene (bac16S) was amplified using a primer pair, 27F and 519R (Lane, 1991; Massana et al., 1997). PCR amplification of the bacterial 16S rRNA gene was carried out with an initial denaturation step at 94°C for 3 min, 25 cycles of amplification (94°C for 1 min, 55°C for 1 min, and 72°C for 1.5 min), and final extension at 72°C for 5 min. Sequencing templates were prepared by pooling three independent PCR amplicons to reduce PCR biases. The nucLSU and bac16S sequences were determined using a 454 GS-FLX Titanium platform (454 Life Sciences, CT, United States) and a PacBio RSII platform (Pacific Bioscience, CA, United States). Sequences were read from both the forward and reverse directions.

### Sequence Processing and Taxonomic Assignment

Sequences obtained with 454 technology were processed to remove primer, linker, and barcode sequences using PyroTrimmer (Oh et al., 2012). In brief, the 3′ end of sequences with low-quality values were trimmed when average quality scores were lower than 20 for a 5-bp window size. Sequences with ambiguous nucleotides or shorter than 300 bp were discarded. Sequence clustering of nucLSU and bac16S sequences was performed by Clustom (Hwang et al., 2013) using 99 and 97% similarity cutoffs, respectively. Chimera detection was conducted using the *de novo* chimera detection algorithm of UCHIME (Edgar et al., 2011). Taxonomic assignment of nucLSU and bac16S sequences was conducted by sequence comparison against reference sequences selected from GenBank nucLSU sequences and the Ribosomal Database Project 16S rRNA training set 16 (Wang et al., 2007).

### Phylogenetic Analysis

Sequences were aligned using ClustalW and manually adjusted (Larkin et al., 2007). Phylogenetic trees were inferred by maximum parsimony (MP), maximum likelihood (ML), and Bayesian analysis. The MP tree was obtained using the tree bisection-reconnection algorithm of MEGA6 (Tamura et al., 2013) with search level five, in which the initial trees were obtained by the random addition of sequences (100 replicates). The ML tree was searched by using PhyML ver. 3.1 (Guindon and Gascuel, 2003) with the GTR evolutionary model and the search options of best tree topology finding by branch swapping of NNIs and SPRs, random addition of sequences (1,000 replicates), and parameter estimation to determine the invariant and transition/transversion ratio. The Bayesian tree was searched by MrBayes ver. 3.2 (Ronquist et al., 2012) with the GTR evolutionary model. Two parallel Markov Chain Monte Carlo runs were performed, each with three heated chains and one cold chain, and the temperature parameter was set to 0.1. Every 100th tree was sampled from 1,000,000 generations of analysis, and a consensus tree was calculated after discarding the first 25% trees as burn-in. The default search conditions were used for other options. Representative sequences of the major operational taxonomic units (OTUs) were deposited at the GenBank database from MN419169 to MN419200 and MK908989 to MK909011.

### Microbial Community Structure and Diversity

The diversity indices, species richness (*S*), Shannon diversity index (*H*′) (Shannon, 1949), and equitability (*J*′) (Pielou, 1966), were calculated for nine groups (Ca, Cm, Cb, Ia, Im, Ib, Ma, Mm, and Mb). Mycobiont sequences were excluded to calculate diversity indices of eukaryotes and 100 sets of randomly selected 100-sequence reads were prepared for each sample. 100 sets of randomly selected 500-sequence reads were prepared for each sample to calculate diversity indices of bacteria.

Bray–Curtis similarity matrices were generated based on standardized relative abundance of algal, LAF, and bacterial OTUs, and relatedness was visualized in non-metric multidimensional scaling (NMDS) ordination plots by the Primer6 (ver. 6.1.16) program (Clarke and Gorley, 2006). A permutational multivariate analysis of variance (PERMANOVA) (Anderson, 2001) with nested design was performed to confirm the variance according to horizontal position (central, intermediate, and marginal) in the lichen colony and vertical position (apical, middle, and basal) in the lichen thalli with 9,999 permutations using PERMANOVA+ in Primer6 (ver. 6.1.16) program (Clarke and Gorley, 2006). Three to five samples were included in each group and samples with less than 1,000 reads were excluded. *Post hoc t*-test was performed to confirm the pair-wise comparisons between the vertical groups (apical, middle, and basal).

### Photochemical Response of Lichen Thalli

The maximum quantum yield of PS II of *C. squamosa* was measured with a Mini-PAM (Walz, Germany). Apical and basal parts of the thalli were positioned in a clip holder and placed in a container with a wet paper towel to maintain high humidity (88–91%) or with silica gel to maintain low humidity (48–56%). Temperature was controlled from 20 to −20°C. The maximum quantum yield of PS II was measured at 20-min intervals.

## Results

### Environmental Conditions

**FIGURE 2:**
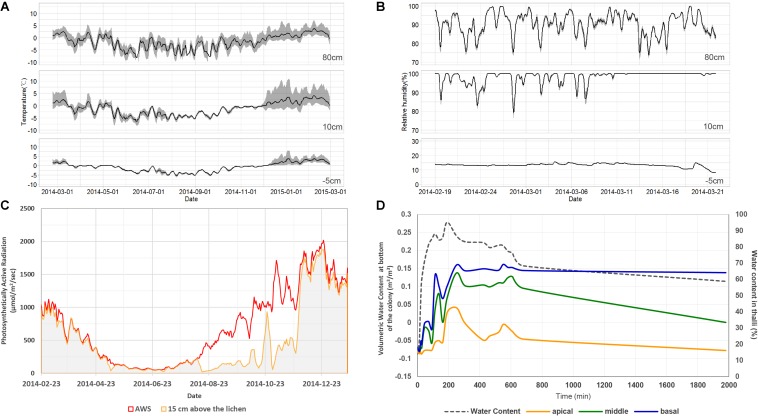
Microenvironments of the habitat of *Cladonia squamosa*. **(A)** Air temperature at 80 and 10 cm aboveground and substrate temperature at 5 cm underground from February 19, 2014 to February 28, 2015. Simple moving average and data range over the past 5 days were represented by the solid line and gray shade, respectively. **(B)** Relative humidity at 80 and 10 cm aboveground and volumetric water content of substrate at 5 cm underground from February 19 to March 21, 2014. Simple moving average and data range over the past 5 days were represented by the solid line and gray shade, respectively. **(C)** Day average of PAR at 80 and 15 cm aboveground from February 19, 2014 to January 20, 2015. **(D)** Water contents at apical, middle, and basal parts of the thalli according to continuous water supply to the substrate and air-dry of the colony in the laboratory.

The average daily air temperature ranged between −15.6 and 5.7°C at a height of 80 cm in 2014 (Figure 2A). Daily variation of air temperature ranged between 0.9 and 13.6°C. The average daily relative humidity ranged between 81.8 and 97.3% at a height of 80 cm during the summer of 2014 (Figure 2B). The air temperature at 10 cm showed lower daily variation during winter season and higher daily variation during summer season than the temperature at 80 cm. Relative humidity at 10 cm was relatively high and less variable compared with that at 80 cm height. The PAR value at 15 cm during winter season was much lower than the PAR value at 80 cm (Figure 2C). A lower PAR value and small daily variation of air temperature at 10 cm imply that the ground was covered with snow during winter. Amplified air temperature variation at 10 cm during summer might be a result of daytime ground heating. The temperature and moisture at 5 cm underground were very stable compared with conditions at 10 or 80 cm aboveground (Figures 2A,B). Light penetration through a lichen colony of *C. squamosa* was very limited when light was projected onto the top of the colony. The average and standard variation were 156.2 ± 6.5 μmol/m^2^/s in apical parts, 25.8 ± 22.5 μmol/m^2^/s in middle parts and 5.6 ± 7.3 μmol/m^2^/s in basal parts. Only 16 and 3% of light could reach the middle and basal parts, respectively. When water was supplied on the substrate, it was transmitted to the basal part of the thalli very quickly and slowly to apical parts (Figure 2D). Maximum water contents after 400 min of water supply were 67, 53, and 24% at the basal, middle, and apical parts, respectively. Moisture at the basal part of thalli was maintained much longer than in the middle and apical parts. These results imply that the basal part of lichen thalli, which is attached to a moss substrate and affected by ground conditions, can maintain very stable temperature and moisture conditions compared with the apical parts, which are affected by sunlight, air temperature, and air humidity.

### Eukaryotic Communities Along the Thallus

Barcoded sequencing of nucLSU produced 1,106–7,094 reads of eukaryotic organisms, including fungi, algae, animal, and protozoa (Supplementary Table S1). The proportion of each taxonomic group was variable according to the vertical position. The mycobiont, *C. squamosa*, was the major component across all samples, representing 52.3–95.2% of all nucLSU sequences. Other components were LAF (1.9–33.0%), green algae (0–14.1%), animals (0–14%), and protozoa (0–1.1%).

**FIGURE 3:**
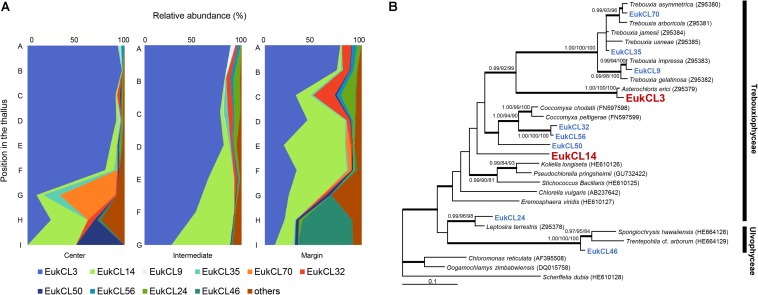
Algal composition along the thalli and phylogenetic tree of the major algal OTUs. **(A)** Relative abundance of 10 major algal OTUs (>0.5% relative abundance). Positions of thalli in the colony and labels of subsection were illustrated in Figure 1B. **(B)** Bayesian tree of 10 major algal OTUs with closely related reference sequences. Thick branches represent those that were conserved in Bayesian, ML, and MP analyses. Posterior probabilities and bootstrap values (>0.9 and >70%) were shown on corresponding branches (Bayesian/ML/MP). The tree was rooted with *Scherffelia dubia* (HE610128, Chlorodendrophyceae). Detailed information for relative abundance of each OTU was included in Supplementary Table S2.

Altogether, 68 green algal OTUs were detected from all samples, and 2–20 algal OTUs were observed in each sample. Out of the 10 algal OTUs with higher than 0.5% in relative abundance, nine OTUs belonged to the class Trebouxiophyceae and one OTU belonged to Ulvophyceae (Figure 3B and Supplementary Table S2). The relative abundance of each algal OTU was variable according to the vertical position within a thallus and several OTUs showed a clear stratification (Figure 3A). The most dominant algal OTU (EukCL3) was closely related to *Asterochrolis erici* of the class Trebouxiophyceae (98.8% similarity of nucLSU) and accounted for up to 97.2% of total algal sequences (Figure 3B and Supplementary Table S2). The relative abundance of EukCL3 was higher at the apical part of the thalli than at the basal part (Figure 3A and Supplementary Figure S2). The second major algal OTU (EukCL14) belonged to the class Trebouxiophyceae with 85.8% sequence similarity with *Koliella longiseta* (Figure 3B and Supplementary Table S2). EukCL14 was rarely recovered from the apical parts of the thalli while it represented high proportion at the middle part of the marginal thallus and basal parts of central and intermediate thalli (Figure 3A, Supplementary Table S2, and Supplementary Figure S2).

**FIGURE 4:**
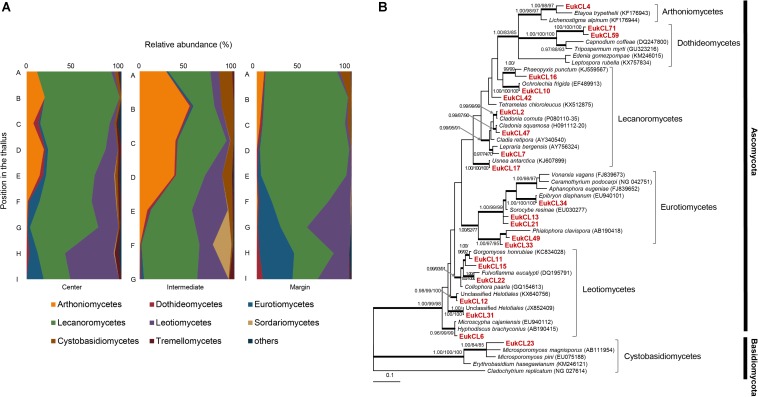
Lichen-associated fungal composition and phylogenetic tree of major fungal OTUs. **(A)** Composition of lichen-associated fungi at the class level along the vertical position of thalli. Positions of thalli in the colony and labels of subsection were illustrated in Figure 1B. **(B)** Bayesian tree of major OTUs (>0.5% relative abundance). Thick branches represent those that were conserved in Bayesian, ML, and MP analyses. Posterior probabilities and bootstrap values (>0.9 and >70%) were shown on corresponding branches (Bayesian/ML/MP). The tree was rooted with *Cladochytrium replicatum* (NG 027614, Chytridiomycota). Detailed information for relative abundance of classes and OTUs was included in Supplementary Tables S3,S4.

Six hundred and forty OTUs of LAF were detected, and each sample contained 2–79 OTUs belonging to seven classes of Ascomycota, three classes of Basidiomycota, and one class of Chytridiomycota (Figure 4A and Supplementary Table S3). Most of the LAF sequences belonged to Lecanoromycetes (22.0–94.2%), Arthoniomycetes (0–52.8%), Leotiomycetes (0–51.3%), and Eurotiomycetes (0–36.3%) (Supplementary Table S3). A high proportion of Arthoniomycetes appeared at the apical and middle parts of the central and intermediate positions. EukCL4 (95.1% similarity with *Etayoa trypethelii*) was the representative OTU of Arthoniomycetes, with high abundance at the apical and middle parts (Supplementary Table S4 and Supplementary Figure S2C). Relative abundance of Lecanoromycetes was higher at the apical and middle parts of central and marginal thalli. None of the representative OTUs of Lecanoromycetes could explain the overall abundance of Lecanoromycetes. Instead, it is thought that many of OTUs are opportunistically distributed, and the sum of all these OTUs form the overall distribution pattern of Lecanoromycetes (Supplementary Table S4). Eurotiomycetes and Leotiomycetes were more abundant at the middle and basal parts of thalli (Figure 4A and Supplementary Table S3).

### Bacterial Communities Along the Thallus

**FIGURE 5:**
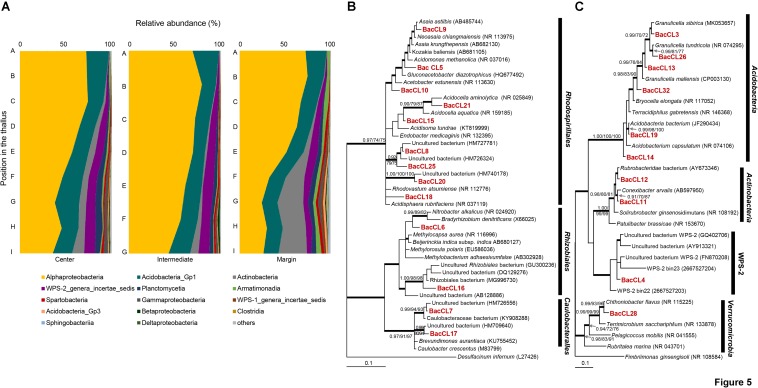
Bacterial composition and phylogenetic trees of major bacterial OTUs. **(A)** Bacterial composition at the class level along the vertical positions of the thalli. **(B)** Bayesian tree of major OTUs of *Alphaproteobacteria*. The tree was rooted with *Desulfacinum infernum* (L27426, *Deltaproteobacteria*). **(C)** Bayesian tree of major OTUs of other bacteria. The tree was rooted with *Fimbriimonas ginsengisoli* (NR_108584, *Armatimonadetes*). Thick branches represent those that were conserved in Bayesian, ML, and MP analyses. Posterior probabilities and bootstrap values (>0.9 and >70%) were shown on corresponding branches (Bayesian/ML/MP). Detailed information for relative abundance of each OTU was included in Supplementary Tables S5–S7.

Barcoded sequencing of 16S rRNA gene produced 1,137–5,002 reads after quality checking (Supplementary Table S1). Plastid and non-bacterial sequences were excluded for further analyses. The proportion of the bacterial sequence reads was highly variable among samples, ranging from 53.3 to 97.3% of total reads (Supplementary Table S1). Bacterial sequences were clustered into 801 OTUs and each sample included from 41 to 315 OTUs. Most of the sequence reads belonged to *Alphaproteobacteria* (24.5–79.5%), *Acidobacteria*_Gp1 (13.3–29%), *Actinobacteria* (0.1–31.1%), candidate division WPS–2 (0.1–11.8%), *Planctomycetia* (0.2–7.6%), *Armatimonadia* (0.1–5.5%), and *Spartobacteria* (0.1–3.3%) (Figure 5A and Supplementary Table S5). *Alphaprotobacteria* and *Acidobacteria*_Gp1 dominated across the samples. Relative abundance of *Alphaproteobacteria* was relatively higher at the apical parts than the basal parts of the thalli (56.0 ± 17.3%) while that of *Acidobacteria*_Gp1 did not show difference along the thalli (19.5 ± 3.8%). *Alphaproteobacteria* was mainly represented by *Rhodospillales* (9.7–54.0%) and followed by *Rhizobiales* (5.7–31.5%) and *Caulobacterales* (1.5–11.2%) at the order level (Supplementary Table S6). *Rhodospirillales* and *Rhizobiales* were consistently major taxonomic groups throughout all positions and abundance was relatively high at the apical and middle parts (Supplementary Table S6 and Supplementary Figure S2). Most of the OTUs that belonged to *Rhodospirillales* were abundant at the apical and middle parts and relatively scarce at the basal parts (Supplementary Table S7). Out of two major OTUs from the order *Rhizobiales*, BacCL6 (94.5% similarity with *Methylorosula polaris*) was abundant at the apical and middle parts of thalli, while BacCL16, which belonged to the lichen-associated *Rhizobiales* (LAR1) lineage, was abundant at the basal part of thalli (Supplementary Table S7 and Supplementary Figure S2). It was related with *Lichenihabitans psoromatis* in the family *Lichenihabitantaceae* (92.3% similarity) (Noh et al., 2019). The proportion of *Caulobacterales* did not change significantly along the thallus profile (Supplementary Table S6). Two major OTUs of *Caulobacterales*, BacCL7 and BacCL17, showed various distribution patterns along the thallus. BacCL7 was distributed throughout the thallus, while BacCL17 was abundant at the middle to basal parts of thalli (Supplementary Table S7 and Supplementary Figure S2). *Actinobacteria* (7.3 ± 9.2%), candidate division WPS-2 (6.6 ± 3.8%), and *Planctomycetes* (3.2 ± 2.3%) showed high relative abundance at the basal parts of thalli (Supplementary Table S5 and Supplementary Figure S2).

### Microbiome Similarity Among Subsamples

The group averaging cluster analysis of algal communities based on the OTU composition revealed that apical and middle parts of thallus formed statistically significant genuine clusters in case of central and intermediate positions (Supplementary Figure S1A). Algal communities of marginal thallus were divided into three clades corresponding to the apical, middle, and basal parts of thalli. LAF communities along the thallus profile were split into two or three clades (Supplementary Figure S1B). Two subsections of the basal parts of thalli at all positions were separately clustered at a significant level (*P* < 0.05). Bacterial communities were clearly divided into two clusters (*P* < 0.01) (Supplementary Figure S1C).

Species richness, Shannon, and equitability diversity indices of eukaryotes were very low at the apical parts compared with the middle and basal parts of thalli (Table 1). Diversity indices of eukaryotes in marginal thalli were relatively high compared with those in the central or intermediate positions in colonies. Diversity indices of bacteria increased from the apical to basal parts of thalli. In contrast to high eukaryotic diversity, bacterial diversity was low in marginal thalli.

**TABLE 1:** Diversity indices of eukaryotic and bacterial communities according to the position.

Position in the colony	Location in the thallus	Eukaryotes						Bacteria					
		Species richness (*S*)		Shannon (*H*′)		Equitability (*J*′)		Species richness (*S*)		Shannon (*H*′)		Equitability (*J*′)	
		Mean	SD	Mean	SD	Mean	SD	Mean	SD	Mean	SD	Mean	SD
Center	Apical	8.12	2.18	0.97	0.21	0.47	0.08	48.4	11.72	2.53	0.04	0.66	0.03
	Middle	17.58	2.67	2.09	0.46	0.73	0.13	99.81	19.77	3.65	0.33	0.79	0.04
	Basal	16.79	4.49	1.81	0.49	0.64	0.11	133.89	19.92	4.21	0.27	0.86	0.04
Intermediate	Apical	7.1	1.69	0.81	0.3	0.42	0.14	57.47	16.3	2.79	0.33	0.7	0.07
	Middle	15.38	6.01	1.63	0.65	0.6	0.15	86.4	9.17	3.42	0.17	0.77	0.02
	Basal	19.25	1.1	2.36	0.11	0.8	0.02	123.97	7.36	4.14	0.07	0.86	0.02
Margin	Apical	9.46	2.15	1.01	0.36	0.45	0.14	38.64	13.14	1.96	0.71	0.53	0.16
	Middle	18.64	3.31	2.07	0.27	0.71	0.05	59.11	15.73	2.92	0.53	0.72	0.09
	Basal	21.38	2.63	2.31	0.19	0.76	0.06	107.2	18.55	3.81	0.25	0.82	0.03

**FIGURE 6:**
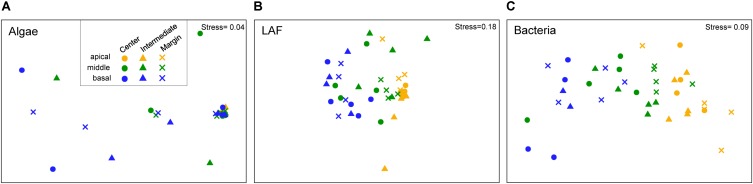
Non-metric multidimensional scaling (NMDS) analysis of microbial community composition based on the OTU composition of algae **(A)**, lichen-associated fungi (LAF) **(B)**, and bacteria **(C)**. Three to five samples with more than thousand sequence reads were included in each group. Standardized OTU abundance matrices were used to calculate Bray–Curtis similarities between samples.

Non-metric multidimensional scaling ordination plots based on the relative abundance of OTUs showed that algal, LAF, and bacterial communities were clustered by the vertical positions of the thallus rather than horizontal positions in the colony (stress = 0.04–0.18) (Figure 6). In particular, apical parts were clearly separated from the other parts. The PERMANOVA analysis revealed that the compositions of algae, LAF, and bacteria in lichen were significantly different according to the vertical position of each group (*P* < 0.02–0.001) (Table 2). Especially, the bacterial community was divided by vertical position with a high *F* ratio (4.958) (Table 2). However, the microbial compositions according to the horizontal positions within the colony were not significantly different (Table 2). Pairwise comparisons among vertical groups were significantly different in all pairs, that is, apical-middle, apical-basal, and middle-basal parts in microbial communities (Supplementary Table S8). The apical-basal pair revealed the highest t statistic value among all algal, LAF, and bacteria communities. In contrast, there was no significant difference among central, intermediate, and marginal positions and these results were similar not only at the OTU level but also at phylum and order levels (data not shown).

**TABLE 2:** Permutational multivariate analysis of variance (PERMANOVA) with nested design according to the horizontal and vertical positions of *Cladonia squamosa.*

Ecological factors	Df	Algae		Lichen-associated fungi		Bacteria	
		*F* ratio	*P*-value	*F* ratio	*P*-value	*F* ratio	*P*-value
Horizontal position	2	0.245	0.8854	0.435	0.8977	0.482	0.741
Vertical position nested in horizontal position	2	2.524	0.0197	2.759	0.0001	4.958	0.0001

### Photochemical Response of Apical and Basal Parts of Thalli

**FIGURE 7:**
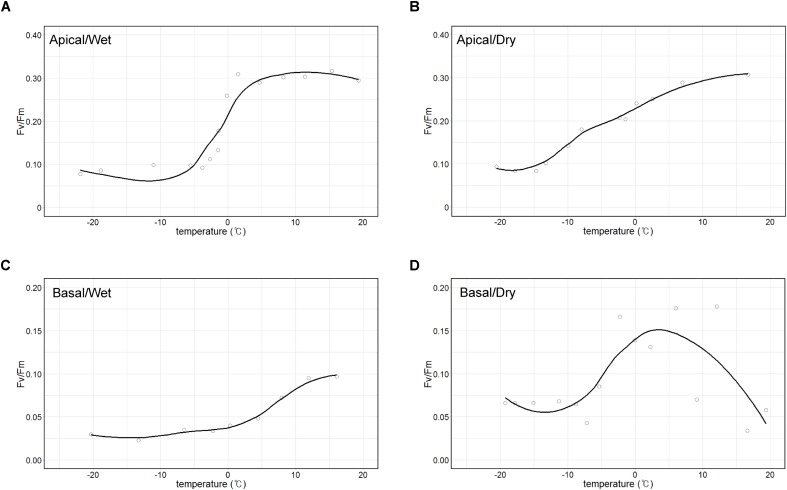
Temperature-response curves of maximum photochemical quantum yield of PS II (*Fv/Fm*). Apical parts in wet **(A)** and dry **(B)** air conditions and basal parts in wet **(C)** and dry **(D)** air conditions.

The maximum quantum yield (*Fv/Fm*) of the apical part of thalli, which is largely composed of *A. erici*, was maintained approximately at 0.3 when temperature was over 0°C and it sharply decreased to 0.1 at freezing temperatures in wet condition (Figure 7A). The value gradually decreased from 0.3 to 0.1, and the minimum value was observed at −15°C in dry condition (Figure 7B). The maximum quantum yield of the basal part according to changing temperature and humidity was quite different from the apical part (Figures 7C,D). The maximum value of *Fv/Fm* was 0.1 and it gradually decreased to 0.025 according to the decrease of the temperature in wet condition. It seemed that photosynthetic molecules were damaged at high temperature in dry condition.

## Discussion

Most of the studies on microbial community in lichens have focused on the macroenvironment such as climate conditions (U’Ren et al., 2012), seasonal patterns (Beck et al., 2014), geographic location, and altitude (Wang et al., 2016) as the lichen microbiome determinants. Since those studies have been performed separately on the photobionts, mycobiont, and other microbial components, a comprehensive understanding on the microbiome of Antarctic lichens is still missing. In this study, we investigated the fine-scale profiling of algal, LAF, and bacterial components through horizontal and vertical sectioning of the Antarctic lichen *C. squamosa* responding to differential microclimatic conditions specifically formed by the three-dimensional structure of a lichen colony.

Microbial diversity showed a clear pattern, with an increase in diversity indices from apical to basal parts of a single thallus and significant differentiation according to the vertical position within a thallus. Vertical stratification of algal, LAF, and bacterial compositions were also presented by chart and ordination analyses (Figures 3–6). This implies that different microenvironment within the thallus may produce the differential lichen microbiome. Several biotic and abiotic factors including physicochemical property within the thallus and colony may produce the different microenvironments in lichen. As *C*. *squamosa* usually grows on mosses (Øvstedal and Smith, 2001; Osyczka and Olech, 2005), water is considered to be more available to the basal part of the thallus in comparison with the apical part, which is exposed to direct sunlight and sudden changes in air moisture (Kranner et al., 2008; Colesie et al., 2012) (Figure 2D). Moreover, the water content of moss measured in the sampling site was kept constant during the same season while the relative humidity aboveground changed dynamically (Figure 2B). Water supply from moss has been suggested as a likely explanation for the greater vitality, including higher growth rates, thicker thalli, and higher net photosynthesis rates, of moss-associated cyanolichen, *Peltigera rufescens*, compared with the vitality of non-moss-associated lichen (Colesie et al., 2012). In addition to the microclimatic conditions, the mycobiont interactions with the mineral soil components could have influence on formation of different microenvironments for the basal parts of *C. squamosa* thalli as the case of saxicolous lichen, where the lower parts of thalli formed specific chemical microenvironments due to the presence of mineral fragments and biomineral crystals (De Los Ríos et al., 2002, 2005). It is also supposed that inoculation of different microbiota from the air or surrounding soil, or anatomical structure and physiological states of host species at different parts can also affect the microbial community, but this issue should be discussed with more specific data. Although it is not possible to specify which microenvironmental factors affected the microbiome at each position, we can make a preliminary conclusion that even the subtle changes of microenvironment found at different vertical positions in a colony could be enough to affect the microbiome composition.

It was observed that all the sub-sections of *C. squamosa* thalli contained diverse algal OTUs, as reported for *C. borealis* and *C. gracilis* (Park et al., 2015), and the composition of each OTU was variable depending on the position in the colony. *Asterochloris erici*, which is known to be resistant to rapid desiccation and rehydration (Gasulla et al., 2013), represented most of the algal component at the apical part of thalli. In contrast, diverse algal OTUs of unclassified genera of Trebouxiophyceae and Ulvophyceae were abundant at the basal part (Figure 3). The apical parts of thalli are exposed to direct sunlight and highly variable temperature and humidity (Figure 2). Compared with the apical parts, the basal parts of thalli were less exposed to sunlight and retained consistent water content owing to the moss substrate (Figure 2). Replacement of major algal OTUs at the basal parts was consistently observed and it is expected that these two algal OTUs may respond differently to various environmental conditions. Photosynthetic responses of photobionts at the apical and basal parts according to variations in temperature and humidity were quite different (Figure 7). Although the current results are not enough to explain their responses specifically according to the microenvironment formed at different positions of lichen thalli, it is proposed that different microclimate provided specific microhabitat for different algal species. The question on the contribution of the second algal OTU to the lichen ecosystem is to be answered in the future researches.

Diverse LAF of Ascomycota and Basidiomycota were observed in *C. squamosa*. EukCL4 belonging to the class Arthoniomycetes was mostly found at the apical and middle parts of thalli and closely related with *E. trypethelii*. *E. trypethelii* is known as a lichenicolous fungus that grows on several corticolous lichens with crustose thalli, such as *Graphidaceae*, *Pertusaria*, and *Lecanora*, in subtropical and tropical regions without causing them any visible damage (Flakus and Kukwa, 2012; Ertz et al., 2014). The species was not observed in the previous study on LAF from several lichens on King George Island, Antarctica (Park et al., 2015). The only report for the closely related species in polar regions was the clonal sequence from Alaska soils (Taylor et al., 2014). Another major LAF OTU, EukCL7, which is related to *Lepraria bergensis*, was highly enriched throughout all parts of marginal thalli (Supplementary Figure S2D). Considering a previous report on the growth of *Lepraria* species on soil, mosses, and other lichens on King George Island (Olech, 2004) and the high portion of *Lepraria* species in the mosses beneath the sampled colony, it is proposed that EukCL7 might have originated from mosses or soil near the colonies of *C. squamosa*. *Ochrolechia frigida*, which is known to overgrow on phanerogams, mosses, and lichens in arctic and alpine regions (Gassmann and Ott, 2000), is capable of saprotrophic nutrition and penetration to substrates using hyphae at the non-lichenized prothallus stage. This may explain the irregular distribution of EukCL10, which is closely related to the *O. frigida* (Supplementary Figure S2). The other major LAF OTUs that are included in Lecanoromycetes were related to the genera *Usnea* (EukCL17), *Cladonia* (EukCL2 and EukCL47), *Phaeopyxis* (EukCL16), and *Tetramelas* (EukCL42). Diverse and frequent recovery of Lecanoromycete LAF raised a question on how they maintain their life on lichen thalli formed by other lichenized fungi (Park et al., 2015). Results in this study also indicate that this question on the life form and relationship among diverse fungal species in lichen thalli remains as an important question in lichen physiology.

Predominance of *Alphaproteobacteria* was observed throughout all parts of the thalli of *C. squamosa*, but the abundance was variable according to the vertical position. *Alphaproteobacteria* was predominant in the apical parts, but the basal parts contained more *Actinobacteria*, candidate division WPS-2, and *Planctomycetes*. The increased abundance of *Actinobacteria* at the basal parts was previously reported by fluorescence *in situ* hybridization in *C. islandica* and *C. arbuscula* (Cardinale et al., 2012). The LAR1 lineage of the order *Rhizobiales* of *Alphaproteobacteria* is known to be one of the most abundant bacterial groups in lichens from temperate regions (Cardinale et al., 2008; Grube and Berg, 2009; Bates et al., 2011; Hodkinson et al., 2012; Printzen et al., 2012), while the order *Rhodosprillales* of *Alphoproteobacteria* dominates lichens in Antarctic areas (Printzen et al., 2012; Park et al., 2016). Consistent with the previous studies, the proportion of *Rhodosprillales* was higher than that of *Rhizobiales* in this study. Nine major OTUs of *Rhodospirillales* were usually more abundant at the apical parts than at the basal parts. Many species of *Rhodospirillales* and *Rhizobiales* are known to fix nitrogen, solubilize phosphate, produce auxin and vitamins, and protect the lichen from stress (Loganathan and Nair, 2004; Pedraza, 2008; Saravanan et al., 2008; Carvalho et al., 2010; Erlacher et al., 2015). These functions may contribute to the survival or growth of the lichens, and thus species with these functions seem to be dominant at the apical part of thalli, which have greater exposure to variable and harsh environments, such as extreme change in the temperature and humidity (Pedraza, 2008; Saravanan et al., 2008).

*Actinobacteria* was abundant at the basal parts of thalli and more than half of all *Actinobacteria* were represented by two major OTUs, BacCL11 and BacCL12, that were closely related to *Conexibacter arvalis* and *Rubrobacteridae*, respectively (Figure 5). *Actinobacteria* in lichen were observed in Lower Devonian lichen *Chlorolichenomycites salopensis* and they are also major soil bacterial components in Antarctic environments (Honegger et al., 2013; Kim et al., 2015, 2019; Van Goethem et al., 2016). The high occurrence of *Actinobacteria* in lichens needs to take into account both long-term relationships with lichen and the influence from soil microbial communities. The proportion of *Actinobacteria* that is frequently recovered from the Alps soils is known to increase when the soil is covered with snow due to lower fluctuations in temperature (Smith et al., 2006; Lauber et al., 2009; Kuffner et al., 2012). The basal parts of *C. squamosa* thalli are affected by the chemical composition of the soil basement and may provide stable physical conditions as the case of the soil that is covered by snow. These physicochemical conditions may explain why *Actinobacteria* is enriched at the basal parts of the lichen thalli.

The candidate division WPS-2 (*Candidatus* Eremiobacteraeota), which was mostly represented by BacCL4, was one of the major bacterial components in the middle and basal parts of thalli (Figure 5). Members of this lineage have been recovered from polluted temperate soil, Greenland ice sheets, Antarctic soil, and mosses (Nogales et al., 2001; Grasby et al., 2013; Stibal et al., 2015; Ji et al., 2016, 2017; Holland-Moritz et al., 2017). Due to the lack of axenic culture representatives of this lineage, physiological characteristics have not been investigated well. However, metagenome sequencing of Antarctic soils suggested that this lineage may ingest atmospheric trace gases to gain sources of energy and carbon (Ji et al., 2017). It is speculative to discuss physiological and ecological roles of BacCL4 because of a lack of knowledge on closely related species and additional information for their activities, but we suggest that it is important to study why it is highly enriched in lichen thalli and what it contributes to the lichen ecosystem, including the possibility of anoxygenic photosynthesis, as proposed for WPS-2 bacteria from boreal mosses (Holland-Moritz et al., 2017).

The comprehensive analysis of microbiome composition of *C. squamosa* collected from Antarctica showed a distinct algal, fungal, and bacterial composition pattern along the thallus profile, implying that each microbe has a preference for a specific microhabitat even within the small thallus and that each may make an intrinsic functional contribution to the lichen ecosystem. To our knowledge, this is the first study that investigated the fine-scale profiling of the algae, fungi, and bacterial compositions in Antarctic lichens. Our results suggest that studies on microbial diversity and its function along the thallus profile in combination with the microclimate characterization will shed light on the adaptation mechanism of *C. squamosa* in one of the most extreme environments on Earth.

## Data Availability Statement

The datasets generated for this study can be found in the GenBank database, MN419169–MN419200 and MK908989–MK909011.

## Author Contributions

H-JN, HL, J-CC, and SH conceived and designed the experiments. H-JN performed the experiments. H-JN and SH analyzed the data. CP contributed to interpreting the results. H-JN, YL, and SH wrote the manuscript. All authors discussed the results and commented on the manuscript.

## Conflict of Interest

The authors declare that the research was conducted in the absence of any commercial or financial relationships that could be construed as a potential conflict of interest.
