# Supplementary material for: Microbiome in Cladonia squamosa Is Vertically Stratified According to Microclimatic Conditions
Source: Front Microbiol. 2020 Feb 25;11:268. doi: 10.3389/fmicb.2020.00268 (PMC7053493; doi:10.3389/fmicb.2020.00268)
Supplement: Supplementary file 1 [file Presentation_1.pdf]

Table S1. Summary of sequencing results

	Center									Intermediate							Margin								
Classification	A	B	C	D	E	F	G	H	I	A	B	C	D	E	F	G	A	B	C	D	E	F	G	H	I
Eukaryotic LSU rDNA sequence reads																									
Fungi																									
Mycobiont	2720	1873	2465	4845	5810	5478	3663	3280	908	1546	4228	2420	3532	2384	4459	3266	6534	1474	1823	1457	780	1147	2967	3474	1611
	(94.4)	(92.1)	(95.2)	(93.3)	(94.1)	(92.3)	(92.3)	(95)	(82.1)	(91.5)	(88.1)	(80.9)	(87.5)	(88.4)	(87.8)	(92.8)	(92.1)	(81.5)	(83.1)	(71.9)	(52.3)	(63.8)	(82.4)	(87)	(86.1)
Lichen associated fungi	55	52	56	133	132	314	200	116	39	43	273	396	262	158	445	131	206	187	210	275	493	467	491	311	126
	(1.9)	(2.5)	(2.2)	(2.6)	(2.1)	(5.3)	(5)	(3.4)	(3.5)	(2.5)	(5.7)	(13.2)	(6.5)	(5.9)	(8.8)	(3.7)	(2.9)	(10.3)	(9.6)	(13.6)	(33)	(26)	(13.6)	(7.8)	(6.7)
Algae																									
Major photobiont	94	106	59	185	186	77	7	7	0	88	241	135	187	89	87	26	273	109	50	126	41	46	23	19	7
	(3.3)	(5.2)	(2.3)	(3.6)	(3)	(1.3)	(0.2)	(0.2)	(0)	(5.2)	(5)	(4.5)	(4.6)	(3.3)	(1.7)	(0.7)	(3.8)	(6)	(2.3)	(6.2)	(2.7)	(2.6)	(0.6)	(0.5)	(0.4)
Minor photobiont	7	3	6	17	31	19	68	22	2	11	50	38	42	46	77	68	77	37	106	159	167	93	70	74	60
	(0.2)	(0.1)	(0.2)	(0.3)	(0.5)	(0.3)	(1.7)	(0.6)	(0.2)	(0.7)	(1)	(1.3)	(1)	(1.7)	(1.5)	(1.9)	(1.1)	(2)	(4.8)	(7.8)	(11.2)	(5.2)	(1.9)	(1.9)	(3.2)
others	0	0	0	0	2	0	0	0	0	0	0	0	0	0	2	1	0	0	0	1	2	0	0	1	0
	(0)	(0)	(0)	(0)	(0)	(0)	(0)	(0)	(0)	(0)	(0)	(0)	(0)	(0)	(0)	(0)	(0)	(0)	(0)	(0)	(0.1)	(0)	(0)	(0)	(0)
Animal	2	0	2	10	7	38	21	23	155	1	4	1	7	11	1	13	3	1	3	2	7	28	20	76	42
	(0.1)	(0)	(0.1)	(0.2)	(0.1)	(0.6)	(0.5)	(0.7)	(14)	(0.1)	(0.1)	(0)	(0.2)	(0.4)	(0)	(0.4)	(0)	(0.1)	(0.1)	(0.1)	(0.5)	(1.6)	(0.6)	(1.9)	(2.2)
Protozoa	2	0	0	0	3	0	1	4	1	0	0	1	4	3	2	4	0	1	2	5	2	15	27	35	21
	(0.1)	(0)	(0)	(0)	(0)	(0)	(0)	(0.1)	(0.1)	(0)	(0)	(0)	(0.1)	(0.1)	(0)	(0.1)	(0)	(0.1)	(0.1)	(0.2)	(0.1)	(0.8)	(0.7)	(0.9)	(1.1)
Unclassified	0	0	0	2	2	11	10	2	1	0	2	2	1	5	8	12	1	0	0	2	0	2	3	4	3
	(0)	(0)	(0)	(0)	(0)	(0.2)	(0.3)	(0.1)	(0.1)	(0)	(0)	(0.1)	(0)	(0.2)	(0.2)	(0.3)	(0)	(0)	(0)	(0.1)	(0)	(0.1)	(0.1)	(0.1)	(0.2)
Total reads	2880	2034	2588	5192	6173	5937	3970	3454	1106	1689	4798	2993	4035	2696	5081	3521	7094	1809	2194	2027	1492	1798	3601	3994	1870
Bacterial 16S rDNA sequence reads																									
Bacteria	2787	2456	2249	2278	2775	1902	3778	1483	1452	1557	845	1842	1493	2612	1934	2634	1343	748	685	2544	3064	3497	1228	3883	1334
	(55.7)	(63)	(74.4)	(76.4)	(77.1)	(89)	(95.9)	(81.6)	(77.6)	(53.3)	(74.3)	(70.1)	(72.6)	(80.3)	(80.6)	(92.3)	(58.7)	(65.4)	(59.2)	(67.8)	(86.9)	(94.5)	(77.6)	(97.3)	(92.9)
Archaea	0	0	0	0	0	0	0	0	1	0	0	0	0	0	0	0	0	0	0	0	0	0	0	0	0
	(0)	(0)	(0)	(0)	(0)	(0)	(0)	(0)	(0.1)	(0)	(0)	(0)	(0)	(0)	(0)	(0)	(0)	(0)	(0)	(0)	(0)	(0)	(0)	(0)	(0)
Plastid	2105	1393	588	419	667	69	37	10	3	1275	229	571	469	257	129	52	901	357	422	1177	408	169	13	44	6
	(42.1)	(35.7)	(19.5)	(14)	(18.5)	(3.2)	(0.9)	(0.5)	(0.2)	(43.7)	(20.1)	(21.7)	(22.8)	(7.9)	(5.4)	(1.8)	(39.4)	(31.2)	(36.5)	(31.4)	(11.6)	(4.6)	(0.8)	(1.1)	(0.4)
Unclassified	111	51	185	286	155	167	125	325	416	88	64	214	93	382	338	167	44	39	50	29	54	33	342	63	95
	(2.2)	(1.3)	(6.1)	(9.6)	(4.3)	(7.8)	(3.2)	(17.9)	(22.2)	(3)	(5.6)	(8.1)	(4.5)	(11.8)	(14.1)	(5.9)	(1.9)	(3.4)	(4.3)	(0.8)	(1.5)	(0.9)	(21.6)	(1.6)	(6.6)
Total reads	5002	3900	3022	2983	3597	2138	3940	1818	1872	2920	1137	2628	2055	3251	2400	2853	2287	1144	1157	3750	3526	3699	1583	3990	1435

Table S2. Relative abundance of major algal OTUs along the thalli

				Center									Intermediate							Margin								
OTU_ID	Class	Close relative	Similarity (%)	A	B	C	D	E	F	G	H	I	A	B	C	D	E	F	G	A	B	C	D	E	F	G	H	I
EukCL3	Trebouxiophyceae	*Asterochrolis erici*	98.8	93.1	97.2	90.8	91.6	85.7	80.1	9.3	24.1	0.0	88.9	82.8	78.0	81.7	65.9	53.0	27.7	78.0	74.7	32.1	44.2	19.7	33.0	24.7	20.4	10.4
EukCL35	Trebouxiophyceae	*Trebouxia jamesii*	99.3	1.0	0.9	0.0	1.0	0.5	3.1	17.3	0.0	0.0	0.0	1.0	4.6	1.3	0.0	0.0	0.0	0.3	2.1	2.6	1.4	1.4	1.4	0.0	1.1	1.5
EukCL9	Trebouxiophyceae	*Trebouxia impressa*	98.3	2.0	0.0	0.0	1.0	0.0	0.0	0.0	0.0	0.0	5.1	0.3	0.6	0.0	0.0	0.0	0.0	0.0	0.0	0.0	0.0	0.0	0.0	0.0	0.0	0.0
EukCL70	Trebouxiophyceae	*Trebouxia asymmetrica*	98.1	0.0	0.0	0.0	0.0	0.0	0.0	57.2	0.0	0.0	0.0	0.0	0.0	0.0	0.0	0.0	0.0	0.0	0.0	0.0	0.0	4.3	3.6	1.1	0.0	0.0
EukCL14	Trebouxiophyceae	*Koliella longiseta*	85.5	1.0	0.0	1.5	3.0	6.5	9.4	6.7	37.9	50.0	0.0	0.3	2.9	6.1	26.7	39.6	60.6	1.4	4.1	15.4	38.9	63.0	51.8	34.4	10.8	19.4
EukCL32	Trebouxiophyceae	*Coccomyxa chodatii*	92	0.0	0.0	3.1	0.5	0.5	1.0	1.3	6.9	0.0	2.0	4.1	5.2	1.7	3.0	0.0	0.0	10.0	6.8	22.4	3.5	1.9	0.0	3.2	0.0	0.0
EukCL50	Trebouxiophyceae	*Coccomyxa subellipsoidea*	87.5	0.0	0.0	1.5	0.0	0.9	1.0	0.0	3.4	50.0	0.0	0.3	0.0	0.0	0.7	0.6	1.1	0.6	0.7	1.9	1.1	0.5	1.4	4.3	3.2	3.0
EukCL56	Trebouxiophyceae	*Coccomyxa chodatii*	91.7	3.0	0.0	0.0	0.5	0.9	0.0	0.0	0.0	0.0	3.0	2.7	1.2	1.3	0.7	0.0	0.0	5.1	3.4	4.5	1.4	0.0	0.0	0.0	0.0	0.0
EukCL24	Trebouxiophyceae	*Leptosira terrestris*	94.4	0.0	0.9	1.5	1.0	1.8	0.0	0.0	0.0	0.0	1.0	6.9	6.4	3.1	1.5	0.6	0.0	4.0	3.4	14.7	2.8	1.4	2.2	0.0	3.2	0.0
EukCL46	Ulvophyceae	*Trentepohlia cf. arborum*	93	0.0	0.0	0.0	0.0	0.9	0.0	0.0	3.4	0.0	0.0	0.0	0.0	0.0	0.0	0.0	2.1	0.0	0.7	0.6	0.7	0.5	2.2	1.1	51.6	56.7
Minor algae				0.0	0.9	1.5	1.5	2.3	5.3	8.1	24.1	0.0	0.0	1.4	1.2	4.8	1.5	6.1	8.5	0.6	4.1	5.8	6.0	7.2	4.4	31.2	9.7	9.0

Major OTUs with higher than 0.5% relative abundance were treated separately and the other OTUs were merged as minor algae. Relative abundance was shaded with gradual dense from red to white.

Table S3. Relative abundance of lichen-associated fungi at the class level along the thalli

		Center									Intermediate							Margin								
Phylum	Class	A	B	C	D	E	F	G	H	I	A	B	C	D	E	F	G	A	B	C	D	E	F	G	H	I
Ascomycota	Arthoniomycetes	10.9	19.2	7.1	18.1	13.6	1.3	0.5	0.0	0.0	27.9	52.8	37.9	36.6	8.2	0.5	0.0	7.7	5.4	3.3	0.0	0.0	0.9	0.2	0.0	0.0
Ascomycota	Dothideomycetes	0.0	0.0	5.4	1.5	3.8	1.3	0.5	2.6	0.0	0.0	1.8	1.8	1.9	3.2	0.9	1.5	1.5	1.1	2.4	1.8	0.8	1.3	1.0	2.9	6.4
Ascomycota	Eurotiomycetes	0.0	0.0	1.8	3.0	3.0	11.2	2.5	8.6	18.0	2.3	2.2	0.5	0.0	0.6	0.5	3.1	0.0	1.1	0.5	1.5	3.7	13.5	23.8	36.3	31.0
Ascomycota	Lecanoromycetes	78.2	78.9	73.2	60.2	50.8	54.5	71.4	27.6	28.2	44.2	22.0	51.5	30.5	43.0	60.9	55.7	74.9	85.6	86.7	94.2	89.8	59.9	27.3	41.5	26.2
Ascomycota	Leotiomycetes	3.6	0.0	5.4	12.8	20.5	25.5	21.1	49.1	51.3	9.3	7.7	3.8	17.9	38.0	13.9	32.8	3.4	0.5	2.9	2.2	5.5	23.0	46.0	16.4	33.3
Ascomycota	Saccharomycetes	0.0	0.0	0.0	0.0	0.0	0.0	0.0	0.0	0.0	0.0	0.0	0.0	0.0	0.0	0.0	0.0	0.0	0.0	0.0	0.0	0.0	0.0	0.2	0.0	0.0
Ascomycota	Sordariomycetes	0.0	0.0	0.0	0.0	0.8	0.6	0.0	0.9	0.0	0.0	0.0	0.3	0.0	0.6	19.6	0.0	0.0	0.0	0.0	0.0	0.0	0.6	0.0	0.0	0.0
Ascomycota	Others	0.0	0.0	1.8	0.0	0.0	0.0	0.0	0.0	0.0	0.0	0.0	0.3	0.0	0.0	0.0	0.0	0.5	0.0	0.0	0.0	0.0	0.0	0.0	1.9	0.0
Basidiomycota	Agaricomycetes	0.0	0.0	0.0	0.0	0.0	0.0	0.0	0.0	0.0	0.0	0.0	0.0	0.4	0.6	0.2	0.0	0.0	0.0	0.5	0.0	0.0	0.0	0.0	0.3	0.0
Basidiomycota	Cystobasidiomycetes	3.6	1.9	1.8	0.8	3.0	1.9	1.0	0.0	0.0	14.0	12.8	3.5	10.3	4.4	1.8	0.8	11.1	3.2	3.8	0.4	0.0	0.2	0.2	0.0	0.0
Basidiomycota	Tremellomycetes	1.8	0.0	1.8	3.0	1.5	2.6	1.0	4.3	2.6	2.3	0.4	0.3	1.5	1.3	0.2	5.3	0.5	0.0	0.0	0.0	0.0	0.2	0.2	0.3	1.6
Basidiomycota	Others	1.8	0.0	1.8	0.0	2.3	0.6	1.0	2.6	0.0	0.0	0.4	0.3	0.8	0.0	0.9	0.0	0.0	0.0	0.0	0.0	0.0	0.2	0.4	0.0	0.0
Chytridiomycota	Chytridiomycetes	0.0	0.0	0.0	0.0	0.0	0.0	0.0	0.0	0.0	0.0	0.0	0.0	0.0	0.0	0.0	0.0	0.0	0.0	0.0	0.0	0.0	0.0	0.0	0.0	0.8
Unclassified	Unclassified	0.0	0.0	0.0	0.8	0.8	0.6	1.0	4.3	0.0	0.0	0.0	0.0	0.0	0.0	0.7	0.8	0.5	3.2	0.0	0.0	0.2	0.2	0.6	0.3	0.8

Relative abundance was shaded with gradual dense from red to white.

Table S4. Relative abundance of major lichen-associated fungal OTUs along the thalli

			Center									Intermediate							Margin								
Phylum	Class	OTU_ID	A	B	C	D	E	F	G	H	I	A	B	C	D	E	F	G	A	B	C	D	E	F	G	H	I
Ascomycota	Arthoniomycetes	EukCL4	10.9	19.3	7.1	18.0	13.6	1.3	0.5	0.0	0.0	25.6	52.7	37.6	36.6	8.2	0.4	0.0	7.8	5.4	3.3	0.0	0.0	0.0	0.2	0.0	0.0
Ascomycota	Dothideomycetes	EukCL59	0.0	0.0	3.6	0.0	2.3	0.0	0.0	0.9	0.0	0.0	0.0	0.8	0.8	0.6	0.4	0.0	0.5	0.0	1.4	1.8	0.2	1.3	1.0	1.0	1.6
Ascomycota	Dothideomycetes	EukCL71	0.0	0.0	1.8	0.0	0.8	1.0	0.5	0.9	0.0	0.0	1.1	0.5	0.4	1.9	0.4	0.8	1.0	1.1	0.5	0.0	0.6	0.0	0.0	0.0	4.0
Ascomycota	Eurotiomycetes	EukCL13	0.0	0.0	0.0	1.5	0.0	1.6	1.0	6.0	15.4	0.0	2.2	0.0	0.0	0.0	0.0	0.8	0.0	1.1	0.5	0.0	1.6	11.1	19.8	32.2	7.9
Ascomycota	Eurotiomycetes	EukCL21	0.0	0.0	0.0	0.0	0.0	7.6	0.0	0.0	2.6	0.0	0.0	0.0	0.0	0.0	0.0	0.0	0.0	0.0	0.0	0.4	0.2	1.3	2.6	1.6	12.7
Ascomycota	Eurotiomycetes	EukCL34	0.0	0.0	0.0	0.0	2.3	0.6	0.0	0.0	0.0	0.0	0.0	0.0	0.0	0.6	0.4	0.8	0.0	0.0	0.0	0.7	0.0	0.0	0.0	1.6	0.8
Ascomycota	Eurotiomycetes	EukCL49	0.0	0.0	0.0	0.0	0.0	0.0	0.5	2.6	0.0	0.0	0.0	0.0	0.0	0.0	0.0	0.0	0.0	0.0	0.0	0.0	0.2	0.2	0.2	1.0	5.6
Ascomycota	Eurotiomycetes	EukCL33	0.0	0.0	0.0	0.0	0.0	0.3	0.0	0.0	0.0	0.0	0.0	0.0	0.0	0.0	0.0	0.0	0.0	0.0	0.0	0.0	0.0	0.0	0.0	0.0	0.0
Ascomycota	Lecanoromycetes	EukCL2	0.0	11.6	1.8	0.0	9.8	16.9	0.5	1.7	0.0	0.0	2.2	0.3	0.0	0.0	0.2	0.0	0.0	0.0	0.0	0.4	0.0	0.0	0.0	2.3	0.0
Ascomycota	Lecanoromycetes	EukCL7	0.0	5.8	33.9	1.5	0.0	9.9	0.0	0.0	0.0	0.0	0.0	1.0	3.1	1.9	47.4	38.4	43.4	49.9	48.6	63.9	44.4	47.7	22.0	30.2	8.7
Ascomycota	Lecanoromycetes	EukCL10	30.9	9.7	0.0	0.0	0.0	6.0	51.6	0.0	0.0	0.0	0.0	0.0	2.7	16.5	0.7	0.0	0.0	5.4	20.5	15.3	28.6	6.6	0.0	0.0	0.0
Ascomycota	Lecanoromycetes	EukCL16	0.0	0.0	0.0	5.3	0.0	0.0	0.0	0.0	0.0	0.0	0.4	0.3	4.6	4.4	0.9	0.8	0.0	0.0	0.5	1.5	0.0	0.0	0.0	0.0	0.0
Ascomycota	Lecanoromycetes	EukCL17	1.8	1.9	1.8	8.3	5.3	1.9	0.0	0.0	0.0	0.0	3.3	0.3	0.4	1.3	0.0	0.0	0.5	0.5	0.0	0.4	0.0	0.0	0.0	0.0	2.4
Ascomycota	Lecanoromycetes	EukCL42	0.0	0.0	0.0	0.0	0.0	0.0	0.0	0.0	0.0	0.0	0.0	40.7	0.4	0.0	0.0	0.0	0.0	0.0	0.0	0.0	0.0	0.0	0.0	0.0	0.0
Ascomycota	Lecanoromycetes	EukCL47	0.0	1.9	1.8	0.0	0.0	0.0	1.6	0.0	0.0	0.0	0.0	0.0	0.4	1.3	0.4	0.0	0.0	0.0	0.0	0.4	0.4	0.2	0.0	0.3	0.8
Ascomycota	Leotiomycetes	EukCL6	0.0	0.0	0.0	0.0	0.0	1.6	3.0	8.6	0.0	0.0	0.4	0.0	0.4	0.0	0.2	0.0	0.0	0.0	0.0	0.0	0.0	2.8	5.3	1.6	10.3
Ascomycota	Leotiomycetes	EukCL11	0.0	0.0	1.8	3.8	9.1	11.5	5.5	19.8	15.4	0.0	0.4	0.3	2.3	8.2	3.6	19.1	1.0	0.0	0.5	0.7	4.1	14.8	18.5	5.5	8.7
Ascomycota	Leotiomycetes	EukCL12	0.0	0.0	0.0	2.3	2.3	1.3	0.0	6.0	17.9	4.7	0.4	0.3	0.4	1.9	0.2	0.0	0.5	0.0	0.5	0.0	0.0	0.2	0.6	0.6	1.6
Ascomycota	Leotiomycetes	EukCL15	1.8	0.0	0.0	0.0	0.0	0.3	0.0	2.6	2.6	0.0	0.0	0.0	0.0	0.0	0.0	0.0	0.0	0.0	0.0	0.0	0.0	3.0	13.0	0.3	0.8
Ascomycota	Leotiomycetes	EukCL22	0.0	0.0	1.8	0.0	0.0	3.2	2.0	0.0	0.0	4.7	5.5	3.0	13.0	24.7	7.6	5.3	0.5	0.0	0.5	0.4	0.0	0.0	0.0	0.0	0.0
Ascomycota	Leotiomycetes	EukCL31	0.0	0.0	0.0	2.3	4.5	3.8	7.0	2.6	2.6	0.0	0.7	0.0	0.4	2.5	0.7	2.3	0.0	0.0	0.0	0.7	0.6	1.3	5.3	4.8	6.3
Basidiomycota	Cystobasidiomycetes	EukCL23	3.6	1.9	1.8	0.8	2.3	1.6	1.0	0.0	0.0	4.7	8.8	3.0	8.8	3.8	1.6	0.8	11.2	3.2	3.8	0.4	0.0	0.2	0.2	0.0	0.0
		Minor OTUs	50.9	47.9	42.9	56.4	47.7	29.6	25.3	48.3	43.6	60.5	22.0	12.1	25.6	22.2	34.6	31.1	33.8	33.4	19.5	13.2	19.0	9.3	11.2	17.0	27.8

Major OTUs with higher than 0.5% relative abundance in lichen-associated fungal sequences were treated separately. Relative abundance was shaded with gradual dense from red to white.

Table S5. Relative abundance of bacterial classes along the thalli

		Center									Intermediate							Margin								
Phylum	Class	A	B	C	D	E	F	G	H	I	A	B	C	D	E	F	G	A	B	C	D	E	F	G	H	I
Acidobacteria	*Acidobacteria*_Gp1	25.1	21.4	17.0	17.3	18.0	22.5	26.6	18.3	21.3	29.0	15.4	16.2	13.3	18.1	18.8	23.7	23.3	18.3	18.4	15.9	19.0	18.2	17.2	17.4	16.7
Acidobacteria	*Acidobacteria*_Gp3	0.0	0.0	0.0	0.0	0.2	0.5	0.8	0.6	0.8	0.0	0.0	0.0	0.1	0.1	0.6	0.8	0.0	0.0	0.0	0.1	0.4	1.9	2.3	2.1	1.3
Actinobacteria	*Actinobacteria*	0.3	0.6	2.3	4.9	7.2	6.2	7.7	7.1	13.1	0.1	0.4	1.0	2.1	1.2	4.0	9.0	0.1	0.6	0.7	2.5	6.0	20.5	28.3	25.5	31.1
Armatimonadetes	*Armatimonadia*	0.7	2.1	0.6	1.3	2.0	1.6	1.4	1.1	1.4	0.1	0.6	0.6	0.6	1.1	0.7	1.0	2.9	2.7	5.0	3.2	1.9	3.5	3.2	3.9	5.5
Bacteroidetes	*Sphingobacteriia*	0.3	0.1	0.0	0.0	0.1	0.1	0.1	0.2	0.3	0.0	0.0	0.0	0.1	0.0	0.0	0.2	0.0	0.2	0.2	0.2	0.5	1.1	0.8	0.9	0.9
Firmicutes	*Clostridia*	0.0	0.0	0.2	0.1	0.0	0.4	0.1	0.1	0.3	0.0	0.1	0.0	0.2	0.0	0.0	0.0	0.0	0.0	0.0	0.1	0.0	0.2	1.4	0.4	0.5
Planctomycetes	*Planctomycetia*	0.2	0.7	1.0	1.9	3.6	6.6	7.1	7.6	5.3	0.2	0.4	1.4	2.7	3.8	3.1	4.7	0.2	0.3	1.5	3.2	4.3	5.1	5.3	3.2	6.0
Proteobacteria	*Alphaproteobacteria*	72.3	73.1	74.1	64.9	56.9	46.3	38.1	45.5	35.7	69.2	79.5	72.1	69.7	61.3	56.1	43.1	72.7	74.9	67.5	63.0	51.5	32.7	24.5	30.1	26.2
Proteobacteria	*Betaproteobacteria*	0.0	0.1	0.1	0.2	0.1	0.3	0.9	1.2	0.8	0.0	0.0	0.1	0.1	0.0	2.4	0.9	0.0	0.0	0.0	0.1	0.0	0.7	1.0	0.8	0.0
Proteobacteria	*Deltaproteobacteria*	0.1	0.0	0.1	0.2	0.2	0.4	0.1	0.2	0.3	0.0	0.0	0.1	0.2	0.5	0.2	0.2	0.2	0.0	0.0	0.5	0.5	0.3	0.5	0.2	0.0
Proteobacteria	*Gammaproteobacteria*	0.0	0.0	0.2	0.2	0.4	1.9	2.8	2.7	4.5	0.0	0.1	0.3	0.1	0.2	1.2	1.8	0.1	0.0	0.2	0.2	1.1	2.2	3.6	1.1	0.3
Verrucomicrobia	*Spartobacteria*	0.1	0.4	0.3	0.8	1.6	1.9	1.6	1.3	2.9	0.1	0.1	1.1	1.0	1.4	1.7	1.8	0.3	0.2	1.1	2.5	2.5	1.7	2.5	2.7	3.3
Candidate division WPS-1	WPS-1_genera_incertae_sedis	0.0	0.2	0.3	0.4	0.8	1.4	1.5	0.5	0.6	0.0	0.3	0.3	0.3	0.5	0.6	0.7	0.1	0.3	0.2	1.1	1.9	1.8	1.9	2.3	1.6
Candidate division WPS-2	WPS-2_genera_incertae_sedis	0.1	1.3	3.6	7.7	8.8	9.1	9.6	11.8	11.7	0.3	2.8	6.9	9.1	11.4	9.5	11.7	0.3	2.4	5.0	6.9	9.5	8.4	5.4	7.3	4.7
Others		1.0	0.2	0.2	0.1	0.3	0.8	1.5	1.7	1.2	1.1	0.0	0.0	0.5	0.2	1.1	0.6	0.0	0.2	0.2	0.4	0.8	1.7	2.2	1.9	2.0

Relative abundance was shaded with gradual dense from red to white.

Table S6. Relative abundance of families of Alphaproteobacteria along the thalli

		Center									Intermediate							Margin								
Order	Family	A	B	C	D	E	F	G	H	I	A	B	C	D	E	F	G	A	B	C	D	E	F	G	H	I
Rhodospirillales	*Acetobacteraceae*	49.7	40.8	40.4	45.2	42.5	32.5	24.2	26.1	20.8	49.7	41.6	41.6	47.0	48.5	42.3	29.1	47.0	54.0	47.5	46.0	35.2	15.4	9.7	11.6	11.8
Rhodospirillales	*Rhodospirillaceae*	0.1	0.1	0.0	0.1	0.0	0.0	0.0	0.0	0.1	0.0	0.0	0.0	0.0	0.0	0.0	0.0	0.0	0.0	0.0	0.0	0.0	0.0	0.0	0.0	0.0
Rhodospirillales	Unclassified	0.0	0.0	0.0	0.0	0.0	0.1	0.0	0.0	0.0	0.0	0.0	0.0	0.0	0.0	0.0	0.0	0.0	0.0	0.0	0.0	0.0	0.0	0.0	0.0	0.0
Rhizobiales	*Beijerinckiaceae*	0.0	0.2	0.1	0.3	1.1	1.2	1.0	0.7	1.4	0.3	0.1	0.1	0.1	0.4	0.9	0.5	0.3	0.3	0.6	0.5	1.0	1.0	0.6	1.4	1.8
Rhizobiales	*Bradyrhizobiaceae*	0.0	0.0	0.0	0.0	0.0	0.0	0.0	0.0	0.3	0.0	0.0	0.0	0.0	0.0	0.0	0.0	0.0	0.0	0.0	0.0	0.0	0.2	0.3	0.4	0.1
Rhizobiales	*Brucellaceae*	0.0	0.0	0.0	0.0	0.0	0.0	0.0	0.0	0.0	0.0	0.1	0.0	0.0	0.0	0.0	0.0	0.0	0.0	0.0	0.0	0.0	0.0	0.0	0.0	0.0
Rhizobiales	*Methylobacteriaceae*	0.0	0.0	0.0	0.0	0.0	0.0	0.0	0.0	0.0	0.0	0.0	0.0	0.0	0.0	0.0	0.0	0.0	0.2	0.0	0.0	0.0	0.0	0.0	0.0	0.0
Rhizobiales	*Rhizobiaceae*	0.0	0.0	0.0	0.0	0.0	0.0	0.0	0.0	0.0	0.0	0.0	0.0	0.0	0.0	1.9	0.0	0.0	0.0	0.0	0.0	0.0	0.0	0.0	0.0	0.0
Rhizobiales	*Roseiarcaceae*	0.0	0.0	0.0	0.0	0.0	0.0	0.0	0.0	0.1	0.0	0.0	0.0	0.0	0.0	0.0	0.0	0.0	0.0	0.0	0.0	0.0	0.1	0.0	0.1	0.0
Rhizobiales	Unclassified	20.9	26.2	23.3	11.5	5.3	5.1	5.2	6.6	5.4	16.5	31.2	23.6	16.1	6.6	6.7	5.2	20.0	13.7	11.9	8.4	8.0	6.8	6.2	6.1	4.6
Caulobacterales	*Caulobacteraceae*	1.5	5.8	10.2	7.6	7.4	6.4	7.3	11.2	6.9	2.7	6.3	6.5	4.0	5.0	3.5	7.4	5.4	6.7	6.9	7.2	6.0	7.3	5.9	7.8	4.5
Rickettsiales	*Rickettsiaceae*	0.0	0.0	0.0	0.0	0.0	0.0	0.0	0.0	0.0	0.0	0.0	0.0	0.0	0.0	0.0	0.0	0.0	0.0	0.0	0.0	0.0	0.0	0.0	0.0	0.0
Rhodobacterales	*Rhodobacteraceae*	0.0	0.0	0.0	0.0	0.3	0.0	0.0	0.1	0.1	0.0	0.0	0.0	2.4	0.0	0.2	0.0	0.0	0.0	0.0	0.0	0.0	0.0	0.0	0.0	0.0
Sphingomonadales	*Erythrobacteraceae*	0.0	0.0	0.0	0.0	0.0	0.0	0.0	0.0	0.0	0.0	0.0	0.0	0.1	0.0	0.0	0.0	0.0	0.0	0.0	0.0	0.0	0.0	0.0	0.0	0.0
Sphingomonadales	*Sphingomonadaceae*	0.1	0.1	0.1	0.2	0.2	0.5	0.2	0.2	0.0	0.0	0.1	0.3	0.1	0.6	0.2	0.7	0.0	0.2	0.0	0.4	1.1	1.5	0.4	0.8	1.6
*Alphaproteobacteria*_incertae_sedis	*Rhizomicrobium*	0.0	0.0	0.0	0.0	0.0	0.0	0.0	0.0	0.3	0.0	0.0	0.0	0.0	0.0	0.0	0.0	0.0	0.0	0.0	0.0	0.0	0.0	0.1	0.0	0.1
*Alphaproteobacteria*_unclassified	Unclassified	0.2	0.1	0.1	0.1	0.0	0.5	0.2	0.7	0.4	0.0	0.0	0.1	0.0	0.2	0.4	0.1	0.1	0.0	0.7	0.4	0.3	0.4	1.2	1.9	1.9

Relative abundance was shaded with gradual dense from red to white.

Table S7. Relative abundance of major bacterial OTUs along the thalli

			Center									Intermediate							Margin								
Class	Order	OTU_ID	A	B	C	D	E	F	G	H	I	A	B	C	D	E	F	G	A	B	C	D	E	F	G	H	I
Alphaproteobacteria	*Rhodospirillales*	BacCL5	10.2	11.6	10.1	11.4	9.7	5.4	3.1	0.6	1.2	10.1	8.1	11.2	14.8	14.8	12.7	5.9	9.9	13.3	14.8	16.3	7.6	0.7	0.4	0.6	0.2
Alphaproteobacteria	*Rhodospirillales*	BacCL8	2.0	4.1	4.7	5.0	4.8	3.8	2.7	3.1	0.4	6.1	8.8	6.1	6.4	8.6	6.2	4.6	6.8	8.1	4.9	5.8	4.0	1.2	0.4	0.3	0.4
Alphaproteobacteria	*Rhodospirillales*	BacCL9	19.6	8.3	5.7	6.0	3.9	2.1	0.7	0.2	0.0	14.2	7.2	6.0	8.2	4.3	2.9	0.9	8.2	6.5	4.5	3.9	2.3	0.4	0.1	0.0	0.1
Alphaproteobacteria	*Rhodospirillales*	BacCL10	0.7	1.7	4.4	5.2	3.9	4.2	3.1	3.9	1.4	2.0	5.5	5.1	5.7	8.4	8.2	5.2	5.9	12.4	7.2	9.0	8.5	2.0	0.6	0.6	1.2
Alphaproteobacteria	*Rhodospirillales*	BacCL15	5.2	2.2	2.3	2.4	3.0	1.5	0.8	1.2	0.4	6.6	4.6	4.2	2.7	1.6	0.8	0.5	3.6	2.7	0.7	0.8	0.3	0.1	0.3	0.1	0.2
Alphaproteobacteria	*Rhodospirillales*	BacCL18	0.3	0.6	0.9	1.6	2.8	2.1	2.2	2.9	2.9	0.1	1.0	0.9	1.2	3.3	2.5	1.7	0.5	1.6	1.9	3.0	2.5	0.9	0.5	0.8	1.6
Alphaproteobacteria	*Rhodospirillales*	BacCL20	8.3	6.9	5.0	4.6	3.4	0.6	0.2	0.2	0.4	4.2	2.4	2.0	0.5	0.7	0.6	0.4	5.3	5.1	5.0	1.7	0.5	0.3	0.6	0.5	0.9
Alphaproteobacteria	*Rhodospirillales*	BacCL21	0.1	0.7	1.0	1.6	2.8	4.0	3.2	1.1	1.1	0.3	0.6	0.6	1.1	1.4	2.3	2.1	0.8	1.0	1.1	1.0	2.5	1.4	0.3	0.3	0.2
Alphaproteobacteria	*Rhodospirillales*	BacCL25	1.2	1.9	3.4	3.7	1.5	1.6	0.4	0.6	0.4	1.6	2.2	2.7	2.9	2.1	1.2	0.7	1.6	1.0	2.2	1.5	1.2	0.5	0.4	0.1	0.0
Alphaproteobacteria	*Rhizobiales*	BacCL6	20.7	26.1	22.7	11.0	3.4	0.8	0.6	0.4	0.0	16.5	30.6	22.7	15.4	4.0	1.1	0.1	19.7	13.2	11.1	5.5	0.8	0.1	0.0	0.1	0.0
Alphaproteobacteria	*Rhizobiales*	BacCL16	0.0	0.1	0.1	0.3	1.9	3.8	3.9	4.9	1.2	0.0	0.4	0.6	0.6	2.6	5.4	4.8	0.2	0.3	0.2	2.6	6.7	5.1	3.4	3.5	2.5
Alphaproteobacteria	*Caulobacterales*	BacCL7	1.5	5.7	10.2	7.1	6.6	3.5	1.9	4.8	3.9	2.7	6.1	6.4	3.8	4.2	2.1	2.4	5.4	6.3	6.5	6.3	3.3	2.2	1.3	0.9	1.5
Alphaproteobacteria	*Caulobacterales*	BacCL17	0.0	0.1	0.0	0.5	0.9	2.7	5.1	5.8	2.5	0.0	0.1	0.1	0.2	0.6	1.3	4.4	0.0	0.2	0.4	0.8	2.5	3.6	2.7	3.5	2.1
Acidobacteria_Gp1	*Granulicella*	BacCL3	11.7	10.8	5.1	4.9	5.5	12.0	15.3	7.9	6.7	10.9	5.8	5.0	5.2	6.1	9.2	14.4	12.2	8.1	5.2	5.4	8.0	6.9	6.5	6.8	5.4
Acidobacteria_Gp1	*Granulicella*	BacCL26	0.1	0.1	0.8	0.5	0.4	0.7	0.7	0.4	0.1	0.5	1.6	3.5	2.9	4.4	2.4	1.4	0.3	0.5	2.4	3.0	1.6	0.6	0.0	1.0	0.8
Acidobacteria_Gp1	unclassified	BacCL13	6.2	6.0	5.6	6.8	4.4	1.4	1.5	0.7	0.1	2.4	3.3	3.2	1.8	1.7	1.4	0.8	5.2	4.6	4.3	2.3	0.8	0.4	0.2	0.7	0.9
Acidobacteria_Gp1	unclassified	BacCL14	0.0	0.0	0.2	0.2	1.3	3.3	3.4	5.2	7.5	0.1	0.0	0.1	0.2	0.5	1.1	1.4	0.0	0.2	0.6	0.4	1.2	3.2	4.3	3.8	3.7
Acidobacteria_Gp1	unclassified	BacCL19	0.0	0.4	2.5	2.6	3.4	3.4	2.5	1.1	0.9	0.0	0.1	1.4	1.8	3.4	2.8	3.3	0.7	1.4	3.7	2.9	3.2	1.9	0.4	0.7	0.7
Acidobacteria_Gp1	unclassified	BacCL32	4.9	2.9	1.6	0.8	1.0	0.1	0.0	0.0	0.0	9.8	2.8	0.7	0.1	0.1	0.0	0.0	3.8	1.9	0.7	0.1	0.1	0.0	0.0	0.0	0.0
Actinobacteria	*Solirubrobacterales*	BacCL11	0.1	0.0	0.4	0.3	0.8	0.7	1.6	1.9	6.7	0.0	0.0	0.0	0.1	0.1	0.4	0.8	0.0	0.0	0.0	0.6	1.3	4.8	13.7	11.6	12.3
Actinobacteria	*Solirubrobacterales*	BacCL12	0.0	0.1	0.8	2.1	3.0	2.3	2.3	3.2	1.9	0.1	0.0	0.3	0.8	0.6	0.9	4.5	0.0	0.2	0.6	0.5	1.8	4.4	5.6	4.2	4.0
WPS-2	WPS-2	BacCL4	0.1	1.3	3.6	7.6	8.4	8.6	8.4	9.9	7.8	0.3	2.8	6.9	9.0	11.3	9.0	10.3	0.3	2.4	4.6	6.6	8.7	6.6	4.0	6.1	3.5
Spartobacteria	incertae sedis	BacCL28	0.0	0.0	0.1	0.6	1.1	1.3	1.2	1.0	2.4	0.0	0.1	0.5	0.8	1.2	1.2	1.3	0.1	0.0	0.2	1.5	1.3	1.1	1.6	1.5	2.0

Major OTUs were selected over the 1% in total bacterial sequence reads. Relative abundance was shaded with gradual dense from red to white.

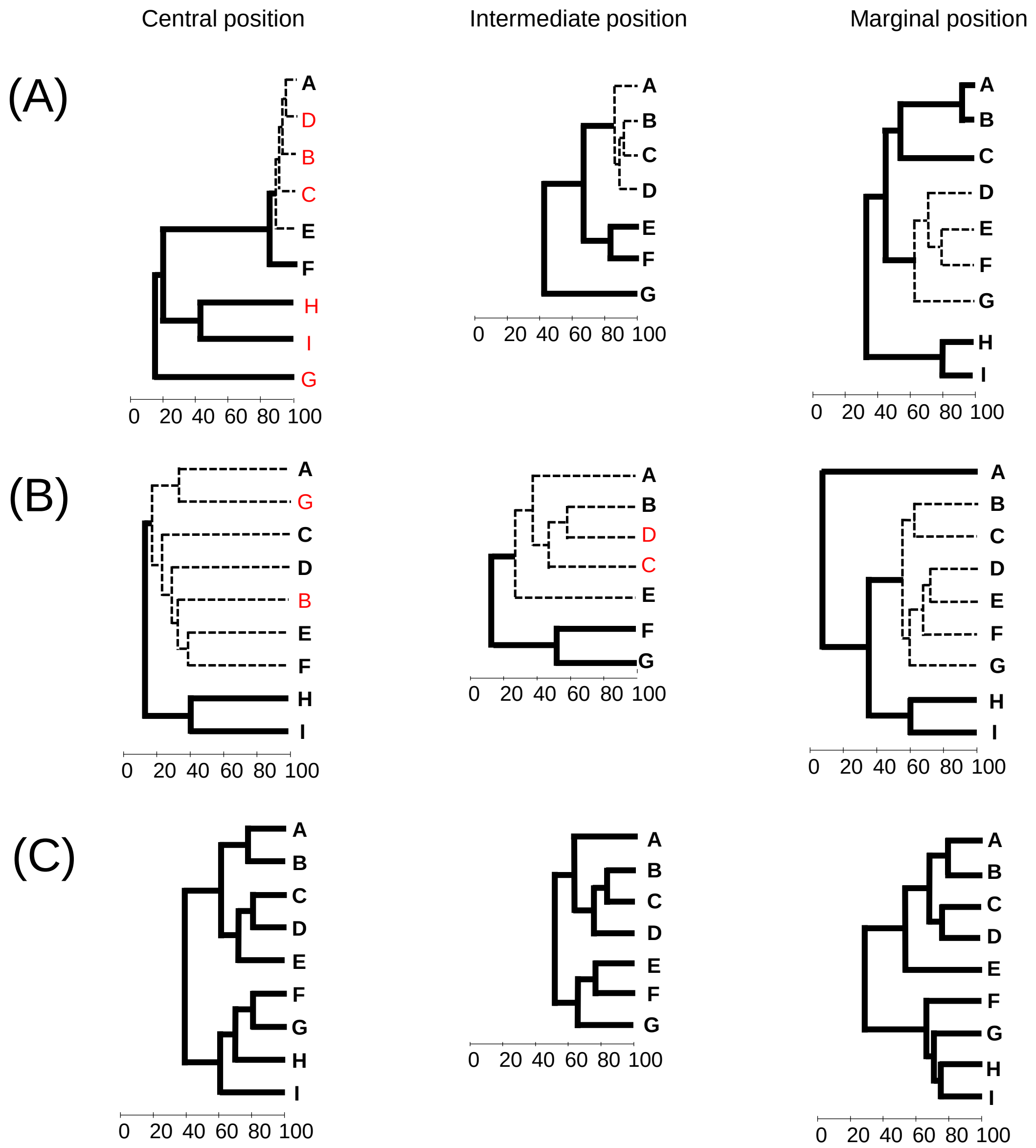

Figure S1. The group averaging cluster analysis of algal (A), lichen-associated fungal (B), and bacterial (C) communities based on Bray-Curtis similarity of OTU abundance. The thick lines indicate significantly supported clusters by the SIMPROF test (P<0.05). Subsamples are labelled from apical to basal parts (A to I) as illustrated in Figure 1B. Black and bold characters denote subsamples that maintain the original order and red characters denote the others.

Table S8. Pair-wise comparisons according to the vertical positions at OTU level

Category	Groups	t	*P-value*	Permutations
Algae	apical-middle part	2	0.0009	9927
Algae	apical-basal part	3.6	0.0001	9914
Algae	middle-basal part	1.9	0.0458	9928
Lichen-associated fungi	apical-middle part	1.8	0.0028	9929
Lichen-associated fungi	apical-basal part	3.2	0.0001	9930
Lichen-associated fungi	middle-basal part	1.7	0.0011	9922
Bacteria	apical-middle part	2.7	0.0003	9936
Bacteria	apical-basal part	4.4	0.0001	9912
Bacteria	middle-basal part	2.5	0.0003	9908

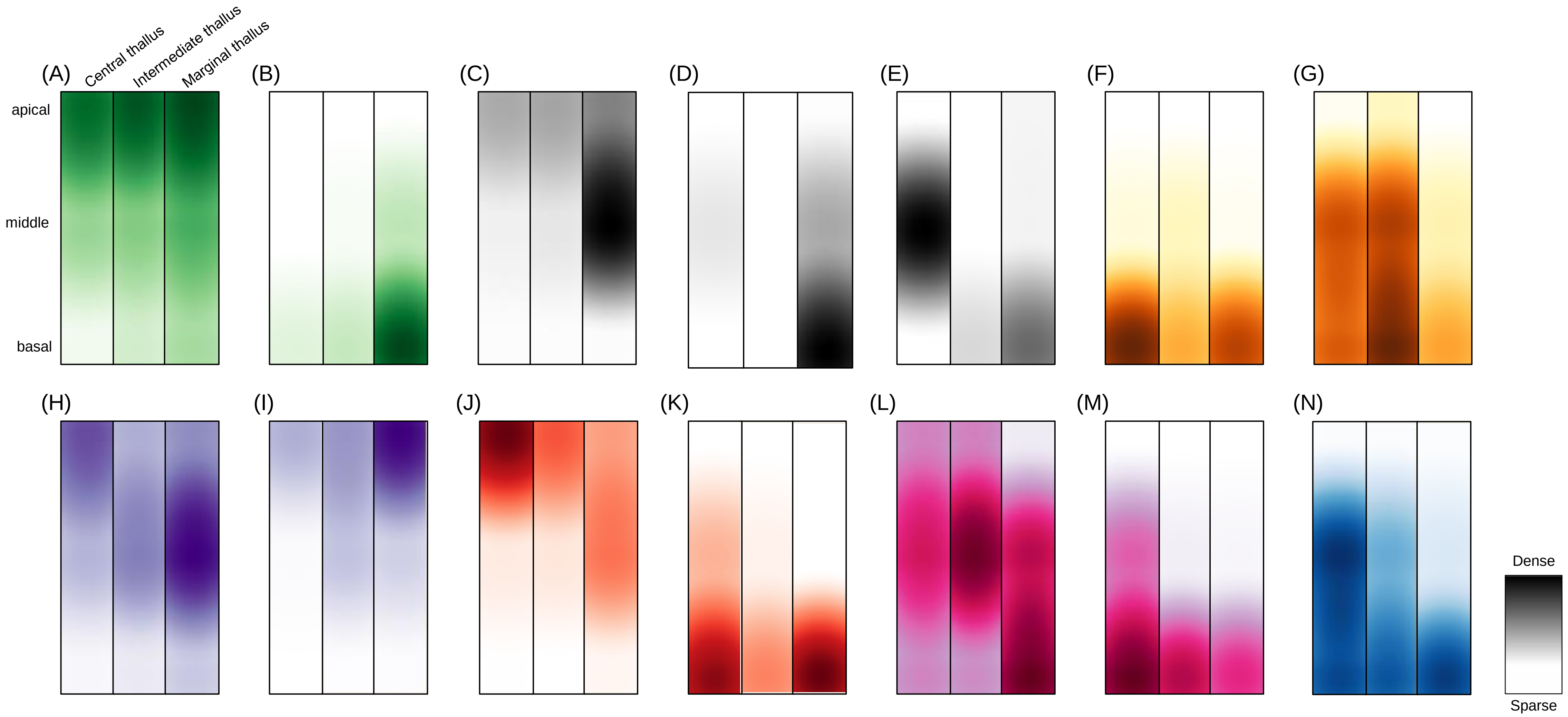

Figure S2. The spatial distribution diagrams of major eukaryotic and bacterial OTUs in *Cladonia squamosa* colony. (A) EukCL3 (*Asterochloris erici*, maximum relative abundance at Ma=0.037); (B) EukCL14 (Trebouxiophyceae, Mb=0.011); (C) EukCL4 (Arthoniomycetes, Mm=0.026); (D) EukCL7 (*Lepraria* species of *Lecanoromycetes*, Mb=0.036); (E) EukCL10 (*Ochrolechia frigida* of Lecanoromycetes, Cm=0.003); (F) BacCL11 (*Actinobacteria*, Cb=0.033); (G) BacCL12 (*Actinobacteria*, Ib=0.045); (H) BacCL5 (*Rhodospirillales*, Mm=0.164); (I) BacCL9 (*Rhodospirillales*, Ma=0.364); (J) BacCL6 (*Rhizobiales*, Ca=0.190); (K) BacCL16 (LAR1 of *Rhizobiales*, Mb=0.014); (L) BacCL7 (*Caulobacterales*, Mb=0.077); (M) BacCL17 (*Caulobacterales*, Cb=0.021); (N) BacCL4 (WPS-2, Cm=0.094).
